# Longitudinal analysis of sociodemographic, clinical and therapeutic factors of HIV-infected individuals in Kinshasa at antiretroviral therapy initiation during 2006-2017

**DOI:** 10.1371/journal.pone.0259073

**Published:** 2021-11-05

**Authors:** Nadine Mayasi Ngongo, Gilles Darcis, Hippolyte Situakibanza Nanituna, Marcel Mbula Mambimbi, Nathalie Maes, Murielle Longokolo Mashi, Ben Bepouka Izizag, Michel Moutschen, François Lepira Bompeka

**Affiliations:** 1 Department of Internal Medicine, Infectious and Tropical Diseases, University Clinics of Kinshasa, Kinshasa, Democratic Republic of the Congo (DRC); 2 Department of Internal Medicine and Infectious Diseases, Liège University Hospital, Liège, Belgium; 3 Biostatistics and Medico-economic Information Department, University Hospital of Liege, Belgium; 4 AIDS reference laboratory, University of Liège, Liège, Belgium; University of Ghana College of Health Sciences, GHANA

## Abstract

**Background:**

The benefits of antiretroviral therapy (ART) underpin the recommendations for the early detection of HIV infection and ART initiation. Late initiation (LI) of antiretroviral therapy compromises the benefits of ART both individually and in the community. Indeed, it promotes the transmission of infection and higher HIV-related morbidity and mortality with complicated and costly clinical management.

This study aims to analyze the evolutionary trends in the median CD4 count, the median time to initiation of ART, the proportion of patients with advanced HIV disease at the initiation of ART between 2006 and 2017 and their factors.

**Methods and findings:**

HIV-positive adults (≥ 16 years old) who initiated ART between January 1, 2006 and December 31, 2017 in 25 HIV care facilities in Kinshasa, the capital of DRC, were eligible. The data were processed anonymously. LI is defined as CD4≤350 cells/μl and/or WHO clinical stage III or IV and advanced HIV disease (AHD), as CD4≤200 cells/μl and/or stage WHO clinic IV. Factors associated with advanced HIV disease at ART initiation were analyzed, irrespective of year of enrollment in HIV care, using logistic regression models.

A total of 7278 patients (55% admitted after 2013) with an average age of 40.9 years were included. The majority were composed of women (71%), highly educated women (68%) and married or widowed women (61%). The median CD4 was 213 cells/μl, 76.7% of patients had CD4≤350 cells/μl, 46.1% had CD4≤200 cells/μl, and 59% of patients were at WHO clinical stages 3 or 4. Men had a more advanced clinical stage (p <0.046) and immunosuppression (p<0.0007) than women.

Overall, 70% of patients started ART late, and 25% had AHD. Between 2006 and 2017, the median CD4 count increased from 190 cells/μl to 331 cells/μl (p<0.0001), and the proportions of patients with LI and AHD decreased from 76% to 47% (p< 0.0001) and from 18.7% to 8.9% (p<0.0001), respectively.

The median time to initiation of ART after screening for HIV infection decreased from 40 to zero months (p<0.0001), and the proportion of time to initiation of ART in the month increased from 39 to 93.3% (p<0.0001) in the same period.

The probability of LI of ART was higher in married couples (OR: 1.7; 95% CI: 1.3–2.3) (p<0.0007) and lower in patients with higher education (OR: 0.74; 95% CI: 0.64–0.86) (p<0.0001).

**Conclusion:**

Despite increasingly rapid treatment, the proportions of LI and AHD remain high. New approaches to early detection, the first condition for early ART and a key to ending the HIV epidemic, such as home and work HIV testing, HIV self-testing and screening at the point of service, must be implemented.

## Introduction

Since 2006, the WHO’s guidelines have been regularly modified regarding the timing of initiation of antiretroviral therapy (ART) for people living with HIV (PLHIV). These recommendations were successively adopted by the national Congolese HIV program.

Initially, the threshold for ART eligibility was set at a CD4 cell count below 200 cells/mm^3^ or a WHO Stage IV clinical condition. The WHO revised their guidelines by increasing the threshold for eligibility for ART in resource-limited settings to a CD4 count at or below 350 cells/mm^3^ in 2010 for all adults living with HIV and then to a CD4 count at/or below 500 cells/mm^3^ in 2013, regardless of WHO clinical internship, giving priority to those with severe or advanced HIV disease (WHO clinical stages III or IV) or a CD4 cell count at or below 350 cells/mm^3^. Finally, since September 2015, all adults diagnosed as positive for HIV infection should initiate ART regardless of CD4 count or clinical stage [1–5]. Since then, some new and updated guidelines have been released to improve the management and care of PLHIV in low- and middle-income countries [6–8].

Efforts to rapidly scale up access to HIV care have been successful at initiating ART in an important number of PLHIV in Sub-Saharan Africa (SSA), where 59.5% of 25.6 million PLHIV underwent ART in 2017 [9]. This led to robust and sustained immune responses and thus to a reduction in the risk of progression to AIDS and the risk of developing non-AIDS-defining illness [10,11].

In contrast, the risk of death is increased in patients with low CD4 status or advanced clinical stage of the disease, mainly during the first few months of antiretroviral therapy [12–23].

Moreover, late enrolled patients in HIV care have an extended chance of transmitting HIV while they are unaware of their HIV infection [11,24]. The multiple negative consequences of late ART initiation for both HIV-infected persons and for transmission of infection are clearly demonstrated [25–27]. This led to progressive guideline modifications toward larger ART eligibility.

In 2017, new guidelines for the management of advanced HIV disease and the rapid initiation of ART were published [8]. Indeed, to achieve the goals of ART, patients must be promptly linked to HIV services, initiate ART, and remain in lifelong care [28]. A significant decrease in the prevalence of advanced disease at ART initiation has been observed due to guideline modifications encouraging earlier ART initiation [29]. However, a significant proportion of patients still initiated ART with advanced HIV disease. Timely ART therapy initiation indeed requires not only early diagnosis of HIV infection but also prompt enrollment and engagement in HIV care.

The Democratic Republic of the Congo (DRC) is a country with a relatively low HIV prevalence (1.2%) [30]. The distribution of this prevalence is heterogeneous. It varies according to age, gender, place of residence, level of education, and civil status, from one province to another. In Kinshasa, the prevalence of HIV is 1.6%. Screening and treatment coverage remain low in DRC. It is estimated that 64% of PLHIV have been diagnosed and that 62% of PLHIV have access to ART [31], although DRC has been scaling up access to HIV care since 2004.

Monitoring the prevalence of advanced HIV infection among people starting ART as well as retention rates in care, adherence to drugs, and viral suppression are necessary to understand the results of the ART program. In this context, the analysis of trends in the prevalence of late initiation of ART and its associated factors is important for improving the effectiveness of HIV testing strategies with a focus on susceptible groups and for assessing early ART implementation programs. Two factors can delay ART initiation. The first is a late diagnosis and presentation for care [32].

The second is delayed treatment initiation following HIV diagnosis.

The objectives of this study were to assess trends in the evolution of median CD4 count and proportion of individuals who initiated ART late or with advanced HIV disease between 2006 and 2017. We also aimed to examine factors associated with advanced HIV disease at ART initiation. We finally studied the evolution of the time between screening for HIV infection and treatment initiation during the same period.

## Methods

### Study design and study population

This retrospective study was based on an open HIV-1 infection treatment cohort in Kinshasa, capital of the DRC. The study population included HIV-positive adults (≥ 16 years old) newly enrolled in HIV care and initiating ART between January 1, 2006 and December 31, 2017 at 25 HIV urban care facilities that provided continuous services during this time period in Kinshasa, capital of the DRC. All subjects received a first-line ART regimen according to Congolese guidelines for the diagnosis and treatment of HIV/AIDS. Patients were included if their CD4+ cell count and/or WHO clinical staging information were available from three months before to one month after ART initiation, given that CD4+ counts are not always performed immediately at enrollment in HIV care or at ART initiation.

The participating facilities are part of national ART programs, and the provision of services at each facility was conducted according to the guidelines of the Congolese Ministry of Health and the WHO. Some programs were receiving technical support during the study period by ICAP at Columbia University, a President’s Emergency Plan for AIDS Relief (PEPFAR) implementing partner and others by Elizabeth Glaser Pediatric AIDS Foundation, through funding from a Global found.

### Data collection

Patient information routinely collected at enrollment in HIV care and during each clinic visit was documented by clinicians or nurses on standard forms used nationally.

Some facilities implemented electronic databases to analyze patient information routinely collected during each visit. For these centers, trained data clerks routinely abstracted relevant data from patients’ medical charts for baseline and follow-up clinic visits and entered them into electronic databases. Data quality assessments were performed every 6 months to assess the completeness and accuracy of data entry.

The data for this study were collected either directly from the standard patient medical records or from the electronic databases, depending on the facility.

Sociodemographic, clinical, and immunological characteristics of all adult PLHIV were examined at ART initiation from 2006 to 2017. The databases used for our study were consulted several times between October 8, 2018, and January 30, 2019.

### Definitions

The main study outcome, which was derived from the routinely collected patient data, was the proportion of HIV-infected individuals who initiated ART at a late stage or with advanced HIV disease.

Late ART initiation was defined as starting therapy while having CD4+ count ≤350 cells/μl and/or WHO clinical stages III or IV. Staging was documented from three months before enrollment and up to one month after ART initiation.

Advanced HIV disease at ART initiation was defined as starting therapy while having a CD4+ count <200 cells/μL or WHO disease stage IV.

A substantial proportion of patients had missing information regarding their CD4+ count or WHO disease stage at ART initiation. These patients were included in our analyses using “missing/unknown” categories for CD4+ count, WHO disease stage, and advanced HIV disease at ART initiation.

### Statistical analysis

Quantitative variables were summarized as the mean standard deviation (SD) or median and interquartile range (IQR: p25—p75). They are presented as frequency tables for the categorized variables (number and percentages). Comparisons of quantitative variables were made using ANOVA or the nonparametric Kruskal–Wallis test when distributions were not Gaussian. Categorized variables were compared using the chi-square test for contingency tables. Comparisons of WHO stage used ordinal logistic regression models.

The impact of AVR starting year on (log-transformed) time between screening and treatment was analyzed using a linear regression model. Logistic regression models were used to study the impact of AVR starting year on treatment <7 days or <1 month after HIV screening. Factors associated with advanced HIV disease at ART initiation were analyzed, irrespective of year of enrollment in HIV care, using logistic regression models.

No missing data has been replaced. The results were considered significant at the uncertainty level of 5% (p <0.05). The statistical analyses were performed using SAS software (version 9.4), and the graphics were made using R software (version 3.5.2).

### Ethical considerations

The Ethics Committee of the School of Public Health, University of Kinshasa approved the use of data for this study (ESP/CE/005/2019). Data were fully anonymized before researchers accessed them, and they were analyzed anonymously and in accordance with ethical rules of confidentiality. As the study was retrospective, there was no need for informed consent for patients, which was lifted by the ethics committee.

## Results

### Sociodemographic, clinical, and immunological characteristics of HIV-infected individuals at ART initiation

The sociodemographic characteristics of the 7278 adults who eventually initiated ART during 2006 and 2017 are presented in Table 1. At ART initiation, more than two-thirds were women (71.1%), and the mean age at ART initiation was 41± 11 years, with 34.4% of PLHIV between 36 and 45 years old. Among those with information on marital status, 36.4% were married, and 24.6% were widowed. The majority of patients (68.6%) had a secondary level of education.

**Table 1:** Sociodemographic status of patients at ART initiation (N = 7278 people living with HIV with ARV treatment initiated between 2006 and 2017 in Kinshasa).

	All	Men	Women	Comparison
	(N = 7278)	(N = 2083)	(N = 5172)	p value
Age (Years), Mean ± SD	40.9 ± 10.6	43.4 ± 10.6	39.8 ± 10.4	<0.0001
15 to 25, N (%)	476 (6.5)	106 (5.1)	370 (7.2)	
26 to 35	1791 (24.6)	336 (16.1)	1453 (28.1)	
36 to 45	2501 (34.4)	714 (34.3)	1781 (34.4)	
46 to 55	1847 (25.4)	657 (31.5)	1179 (22.8)	
≥ 56	663 (9.1)	270 (13.0)	389 (7.5)	
*Total*	*7278 (100*.*0)*	*2083 (100*.*0)*	*5172 (100*.*0)*	
Marital status, N (%)				<0.0001
Married	529 (36.4)	244 (58.6)	280 (27.6)	
Divorced	171 (11.8)	27 (6.5)	141 (13.9)	
Widowed	358 (24.6)	39 (9.4)	307 (30.2)	
Single with child	171 (11.8)	15 (3.6)	156 (15.4)	
Single without children	224 (15.4)	91 (21.9)	131 (12.9)	
*Total*	*1453 (100*.*0)*	*416 (100*.*0)*	*1015 (100*.*0)*	
Level of studies, N (%)				<0.0001
Illiterate	26 (2.1)	1 (0.3)	24 (2.8)	
Primary school	135 (11.2)	15 (4.4)	115 (13.6)	
Secondary school	829 (68.6)	208 (61.6)	609 (71.7)	
Technical/Professional	0 (0.0)	0 (0)	0 (0)	
University	219 (18.1)	114 (33.7)	101 (11.9)	
*Total*	*1209 (100*.*0)*	*338 (100*.*0)*	*849 (100*.*0)*	

Table 2 summarizes the clinical and immunological characteristics of our cohort. Patients initiated ART with a mean weight of 56 ±12 kg and a mean BMI of 20.4± 4.4. 32% suffered from undernutrition defined as a BMI < 18.

**Table 2:** Clinical and immunological status of patients at ARV treatment initiation (N = 7278 people living with HIV with ARV treatment initiated between 2006 and 2017 in Kinshasa).

	All	Men	Women	Comparison
	(N = 7278)	(N = 2083)	(N = 5172)	p value
Weight (kg), Mean ± SD	55.7 ± 12.2	57.3 ± 10.9	55.0 ± 12.6	<0.0001
	(N = 3713)	(N = 1100)	(N = 2693)	
Height (cm), Mean ± SD	164 ± 7.6	170 ± 7.4	162 ± 6.6	<0.0001
	(N = 1152)	(N = 320)	(N = 812)	
BMI (kg/m^2^), Mean ± SD	20.4± 4.4	20.1± 3.7	20.4± 4.6	0.28
Undernutrition (< 18), N (%)	284 (32.1)	79 (33.2)	200 (31.8)	
Normal (18–24.9)	489 (55.2)	139 (58.4)	339 (53.9)	
Overweight (25–29.9)	84 (9.5)	16 (6.7)	67 (10.6)	
Obesity (≥ 30)	28 (3.2)	4 (1.7)	23 (3.7)	
*Total*	*885 (100*.*0)*	*238 (100*.*0)*	*629 (100*.*0)*	
ARV treatment starting year, N (%)				
2006	731 (10.0)	192 (9.2)	522 (10.1)	
2007	653 (9.0)	177 (8.5)	472 (9.1)	
2008	267 (3.7)	82 (3.9)	183 (3.6)	
2009	84 (1.1)	25 (1.2)	59 (1.1)	
2010	237 (3.3)	49 (2.3)	188 (3.6)	
2011	256 (3.5)	70 (3.4)	186 (3.6)	
2012	372 (5.1)	117 (5.6)	255 (4.9)	
2013	674 (9.3)	191 (9.2)	483 (9.4)	
2014	805 (11.1)	243 (11.7)	562 (10.9)	
2015	753 (10.3)	237 (11.4)	516 (10.0)	
2016	1131 (15.5)	328 (15.7)	803 (15.5)	
2017	1315 (18.1)	372 (17.9)	943 (18.2)	
*Total*	*7278 (100*.*0)*	*2083 (100*.*0)*	*5172 (100*.*0)*	
Time between HIV screening and ARV treatment initiation (days), Median (IQR)	12 (0–43)	12 (0–44)	11 (0–42)	0.88
	(N = 5846)	(N = 1699)	(N = 4128)	
WHO Stage, N (%)				0.046
I	1012 (18.0)	273 (16.5)	738 (18.7)	
II	1290 (22.9)	379 (22.9)	903 (22.9)	
III	2958 (52.6)	881 (53.3)	2066 (52.3)	
IV	364 (6.5)	119 (7.2)	242 (6.1)	
*Total*	*5624 (100*.*0)*	1652 (100.0)	3949 (100.0)	
CD4 (Nb/mm^3^), *Median (IQR)*	213 (110–337)	203 (98–317)	216 (116–350)	0.0007
<100, N(%)	299 (11.3)	94 (11.9)	204 (11.0)	
100–199	918 (34.8)	291 (37.0)	625 (33.8)	
200–349	809 (30.6)	249 (31.6)	557 (30.1)	
350–499	325 (12.3)	84 (10.7)	241 (13.0)	
≥ 500	290 (11.0)	69 (8.8)	221 (12.0)	
*Total*	*2641 (100*.*0)*	787 (100.0)	1848 (100.0)	
Late ART initiation ^§^, N (%)	4130 (69.7)	1248 (71.2)	2865 (69.0)	0.093
	(N = 5925)	(N = 1752)	(N = 4150)	
Patients initiating ART with advanced disease ^¶^, N (%)	1497 (25.3)	471 (26.9)	1020 (24.6)	0.063
	(N = 5925)	(N = 1752)	(N = 4150)	

^§^ Late ART initiation: patients starting ART with CD4 T cells <350/mm^3^ or WHO stage III or IV.

^¶^ ART initiation with advanced HIV disease: patients starting ART with CD4 T cells <200/mm^3^ or WHO stage IV.

Among PLHIV who initiated ART, only 2641 had information on CD4+ (36.3%), and their median CD4+ count was 213 cells/μL (IQR, 110–337). A total of 5624 patients (77.3%) had WHO disease stage information at ART initiation, with 59% of patients with WHO disease stage III or IV disease.

Between 2006 and 2017, women started ART at a WHO clinical stage slightly lower than men (p = 0.046). Accordingly, men had more advanced immunodeficiency than women at ART initiation (p = 0.0007).

As indicated in Table 2, 4130 patients (69.7%) were treated late, and 1497 (25.3%) had advanced HIV disease at ART initiation.

According to clinical versus immunological criteria, 59.1% vs. 76.7% were classified as treated late, and 6.5% vs. 46.1% of PLHIV started ART with advanced HIV disease, respectively. The median time between HIV screening and ART initiation was 12 months (IQR 0–43) (Table 2).

### Evolution of the therapeutic regimens of PLHIV at ART initiation

**Fig 1:**
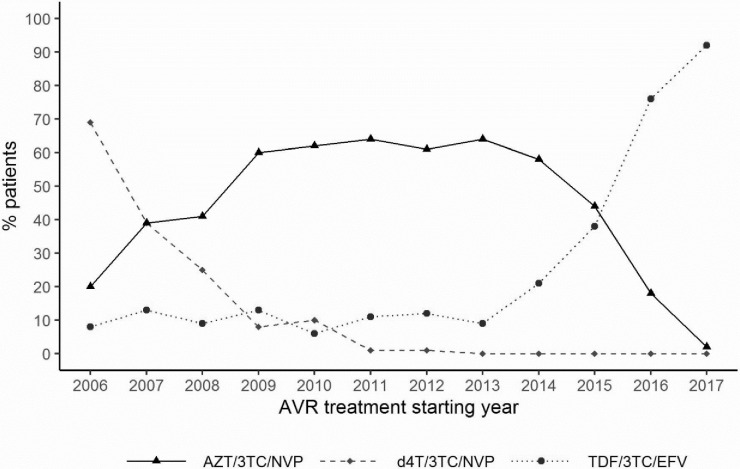
Evolution of the first-line ART regimen preferentially recommended by the WHO over time.

Throughout the study period, the combination of two nucleoside reverse transcriptase inhibitors (NRTIs) and one nonnucleoside reverse transcriptase inhibitor (NNRTI) was almost 98.4% on average. In 2006, the most prescribed regimen was d4T/3TC/NVP. From 2008, AZT/3TC/NVP was the regimen of choice until 2015. From 2014, the regimen containing TDF gradually increased and doubled between 2015 and 2016 (38% vs. 76%). In 2017, almost all (92%) of the PLHIV initiating ART received TDF/3TC/EFV. The gradual abandonment of d4T in favor of AZT or TDF was almost complete in 2011 (Table 3). Fig 1 shows the evolution of preferential treatment regimens recommended by the WHO and adopted by the national HIV control program in the DRC over time.

**Table 3:** ART regimen (at ART initiation) (N = 5807), N(%).

Treatment	2006	2007	2008	2009	2010	2011	2012	2013	2014	2015	2016	2017
	N = 172	N = 61	N = 44	N = 62	N = 230	N = 223	N = 343	N = 669	N = 805	N = 753	N = 1130	N = 131
D4T/3TC/EFV	2 (1)	0 (0)	1 (2)	1 (2)	4 (2)	1 (1)	0 (0)	0 (0)	0 (0)	0 (0)	0 (0)	0 (0)
D4T/3TC/NVP	117 (69)	24 (39)	11 (25)	5 (8)	23 (10)	2 (1)	3 (1)	1 (0)	1 (0)	0 (0)	0 (0)	2 (0)
AZT/3TC/EFV	4 (2)	3 (5)	2 (5)	4 (7)	34 (15)	41 (18)	76 (22)	152 (23)	144 (18)	87 (12)	37 (3)	14 (1)
AZT/3TC/NVP	34 (20)	24 (39)	18 (41)	37 (60)	142 (62)	143 (64)	209 (61)	425 (64)	470 (58)	328 (44)	198 (18)	31 (2)
TDF/3TC/EFV	13 (8)	8 (13)	4 (9)	8 (13)	14 (6)	25 (11)	42 (12)	60 (9)	171 (21)	286 (38)	857 (76)	1205 (92)
TDF/3TC/NVP	1 (0)	2 (4)	5 (11)	1 (2)	4 (2)	8 (4)	8 (2)	20 (3)	13 (2)	40 (5)	23 (2)	39 (3)
Other	1 (0)	0 (0)	3 (7)	6 (8)	9 (3)	3 (1)	5 (2)	11 (1)	6 (1)	12 (1)	15 (1)	24 (2)

### Trends in median CD4+ count among patients at ART initiation and trends in median time between HIV screening and ART initiation

**Fig 2:**
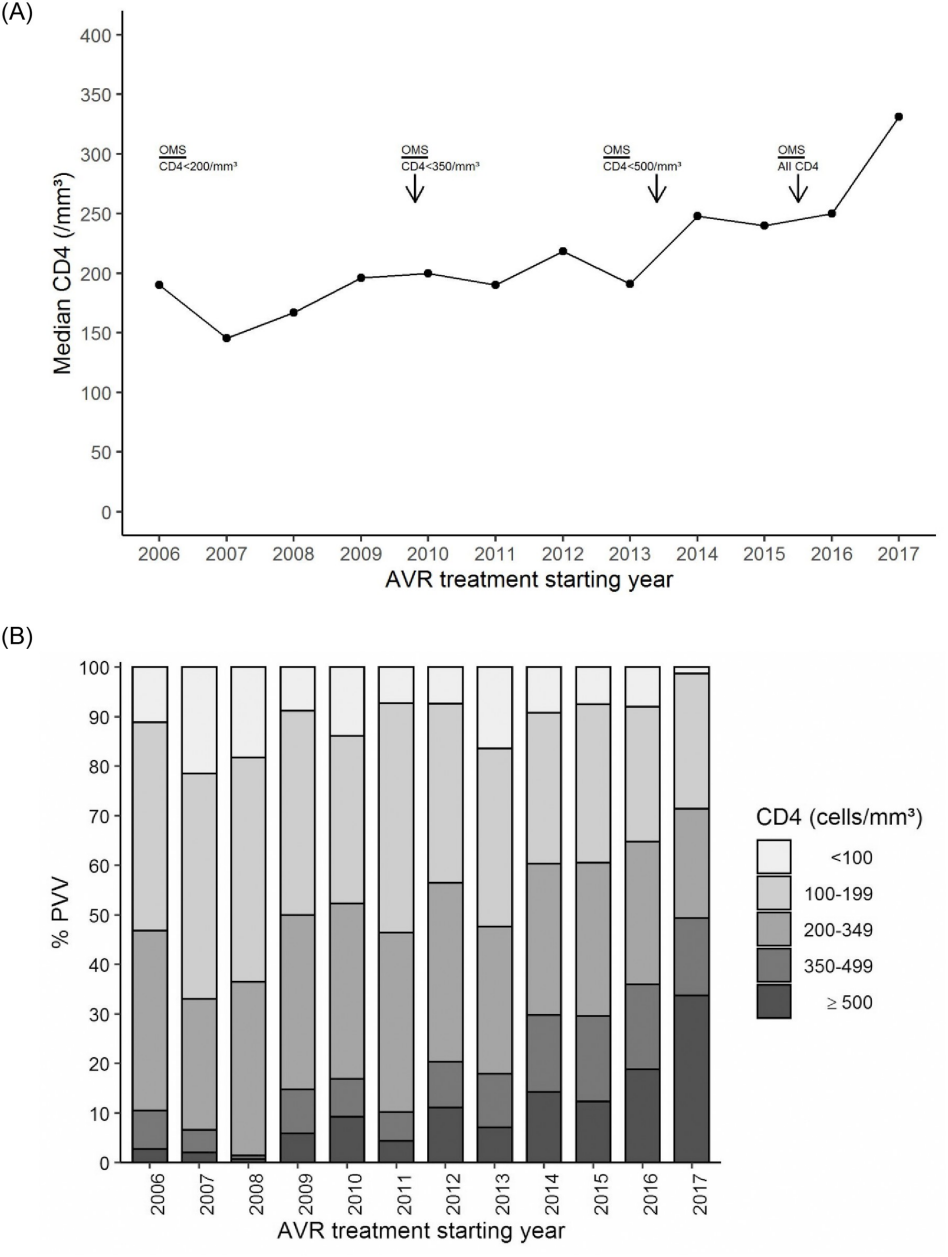
A. Evolution of median CD4 with AVR treatment starting year. B. Evolution of the proportion of patients by CD4 categories with ART starting year.

Between 2006 and 2017, a significant increase in median CD4+ count was noted among patients who started ART (from 190/mm^3^ in 2006 to 331/mm^3^ in 2017) (p<0.0001). Considering the year following new recommendations regarding the threshold CD4 count of eligibility for antiretroviral treatment, the median rate of CD4 increased significantly from 190 in 2011 to 248 in 2014 (p<0.0001) and then from 250 in 2016 to 331 in 2017 (p<0.0001) (Fig 2A).

During the period of study, a significant decrease in time between HIV screening and ARV treatment initiation was observed (from 40 months in 2006 to 13 months in 2015 and less than 1 month in 2017, p<0.0001). Thirty-nine percent of PLHIV initiated ART within one month of their screening in 2006, compared to 93.3% in 2017. The proportion of treated patients within 7 days of HIV screening increased from 14% to 83% between 2006 and 2017 (p <0.0001) (Table 4).

**Table 4:** Evolution of median time between HIV screening and ART initiation, % patients treated within time with ARV treatment initiation year.

ARV treatment starting year	N	Median (IQR) time between HIV screening and ARV treatment initiation (days)	% patients treated <7 days after HIV screening	% patients treated <1 month after HIV screening
2006	510	40 (13–183)	14.3	39.0
2007	565	32 (10–132)	19.6	45.2
2008	234	47 (25–172)	17.1	33.6
2009	61	50 (8–281)	22.2	36.5
2010	91	31 (1–114)	25.6	43.8
2011	110	29 (7–90)	23.2	49.0
2012	211	27 (6–61)	27.5	55.3
2013	480	19 (6–38)	29.8	66.6
2014	637	21 (3–41+)	32.4	65.1
2015	636	13 (0–32)	41.4	73.7
2016	843	1 (0–6)	63.9	85.2
2017	1107	0 (0–0)	83.4	93.3
Evolution				
Coef (SE)	-0.28 (0.0074)		0.29 (0.0097)	0.23 (0.0079)
p value	<0.0001		<0.0001	<0.0001

### Changes in the proportion of patients initiating ART with advanced HIV disease over time

**Fig 3:**
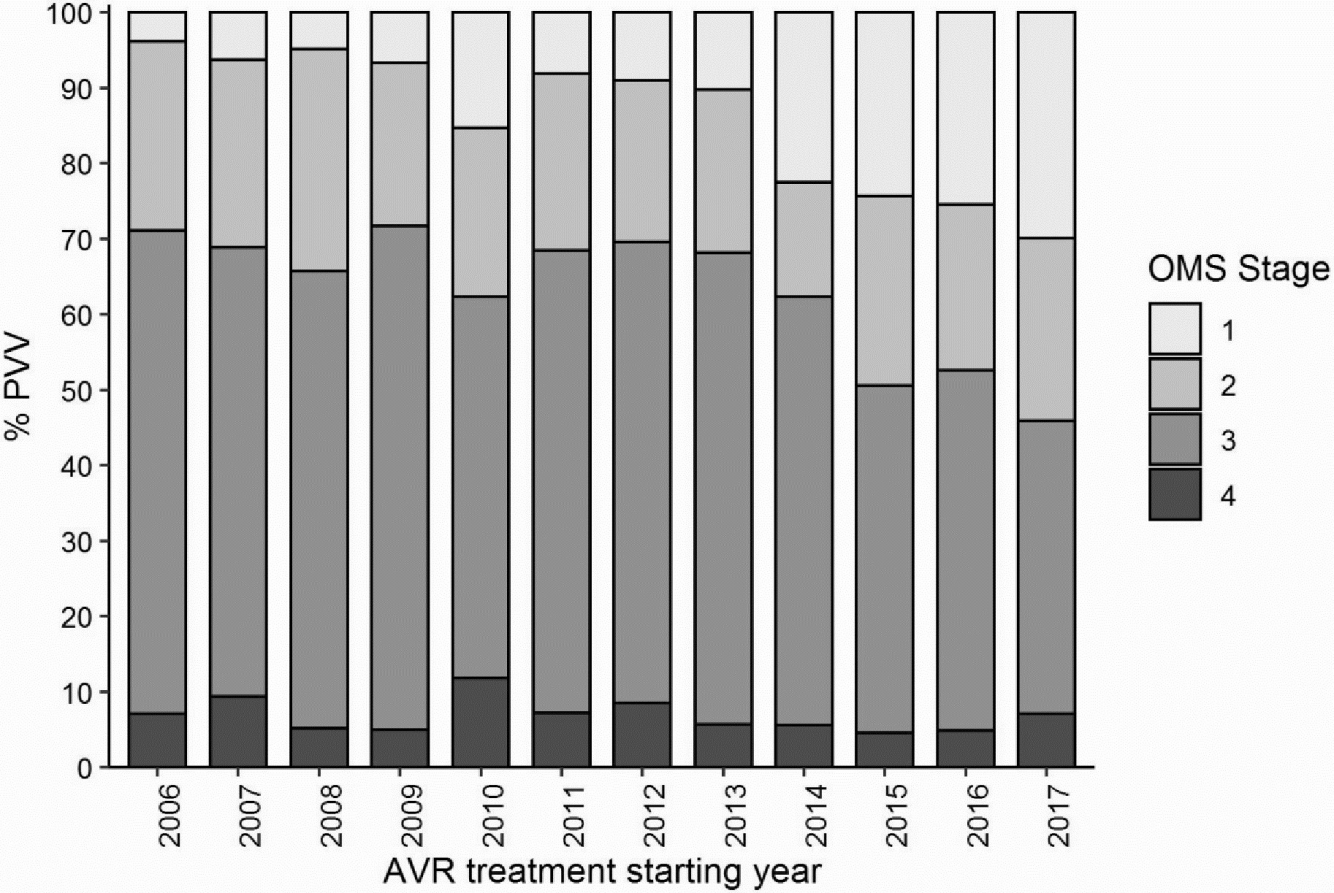
Evolution of the proportion of patients by OMS stage with AVR treatment starting year.

**Fig 4:**
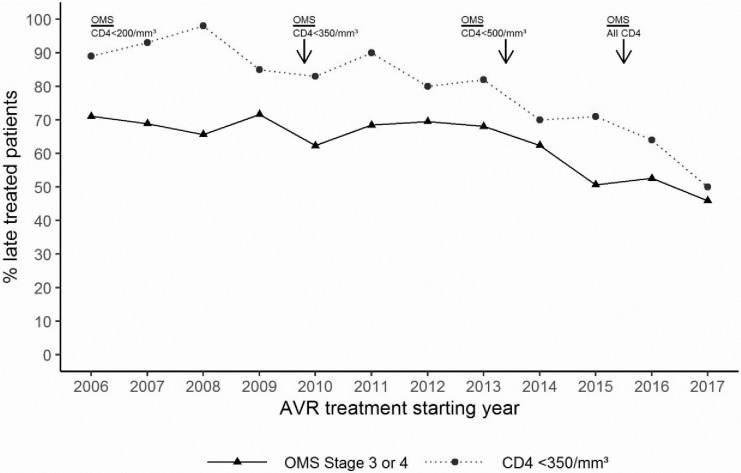
Proportion of patients initiating ART late overtime.

**Fig 5:**
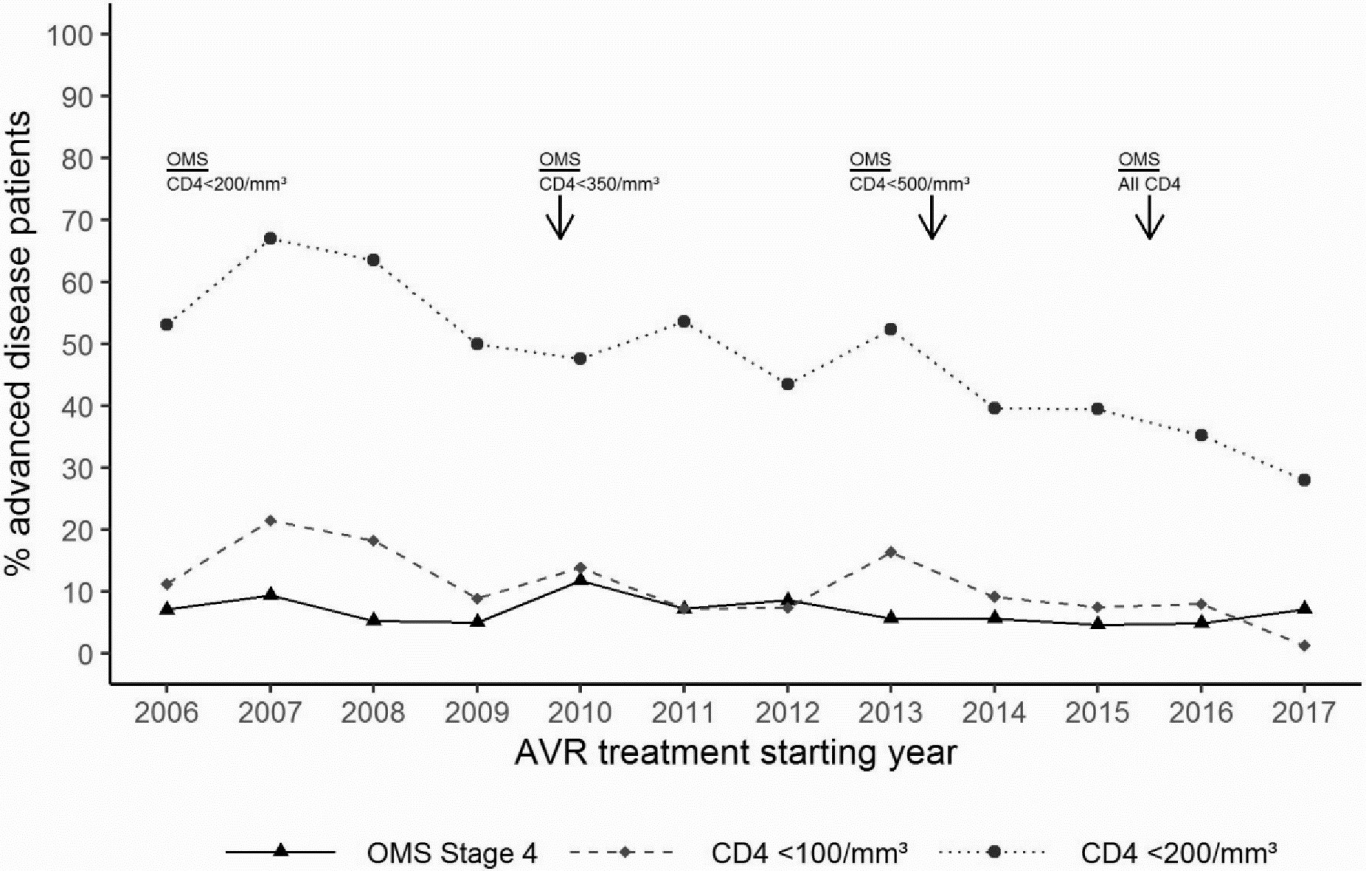
Proportion of patients initiating ART with advanced HIV disease over time.

Figs 2B and 3 describe the evolution of the distribution of patients according to their WHO clinical stage or their immunological status at ART initiation. The proportion of patients treated late with or without advanced HIV gradually decreased year after year. The proportion of patients treated late (CD4+ ≤350 cells/μL) decreased between 2006 and 2017 from 89% to 50%, and the proportion of PLHIV treated with advanced HIV disease (CD4+ <200 cells/μL) decreased from 53% to 28% (p< 0001) (Figs 4 and 5).

**Fig 6:**
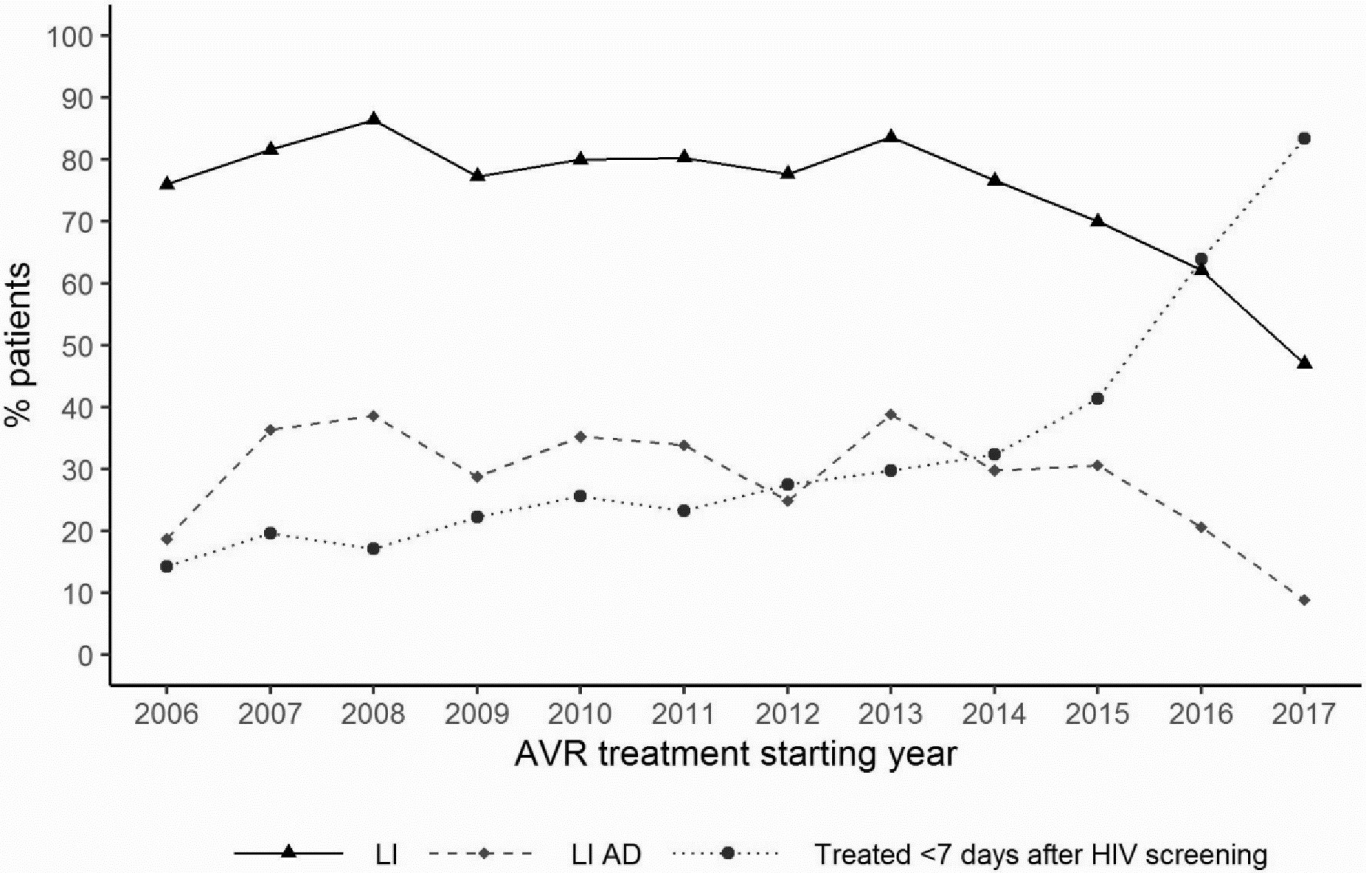
Proportion of patients who started treatment late according to CD4 and WHO stages and of patients treated within 7 days of diagnosis.

Considering the different periods of this cohort according to the eligibility criteria for ARVs, the proportion of patients who initiated ART late (CD4≤350 cells/μL) decreased significantly, by 10% between the first and second periods (p<0.0001) and 13% (p<0.0001) for the following periods. Between the first and last periods, the decline in this proportion was 32% (p<0.0001). The trend in the proportion of patients initiating ART with advanced HIV disease (CD4≤200 cells) declined by 21% (p<0.0001%), 13% (p<0.025) and 18% (p<0.0016) during the three periods, respectively. Between the first and last periods, the decline in these proportions was 44% (p<0.0001) (Table 5). Interestingly, the reduction in the proportion of patients treated late both with and without advanced HIV disease was more pronounced from 2016, the year of implementation of the "test and treat" strategy (Fig 6).

**Table 5:** Evolution of median CD4 cell count at ART initiation and OMS recommendations.

	% late treated				
	Median CD4 (/mm^3^)	WHO Stage IV	CD4<200/mm^3^	WHO Stage III or IV	CD4<350/mm^3^
I. Jan 06–Oct 09	164	7.7	62.1	69.4	93.1
II. Nov 09–May 13	201	7.7	49.3	66.5	83.6
III. Jun 13–Aug 15	235	5.4	42.7	60.8	72.8
IV. Aug 15–Dec 17	254	5.9	34.9	48.5	63.7
Δ II–I (%)	+23%	0%	-21%	-4%	-10%
	p<0.0001	p = 0.99	p<0.0001	p = 0.19	p<0.0001
Δ III–II (%)	+17%	-30%	-13%	-9%	-13%
	p = 0.0002	p = 0.044	p = 0.025	p = 0.015	p<0.0001
Δ IV–III (%)	+8%	+9%	-18%	-20%	-13%
	p = 0.0007	p = 0.55	p = 0.0016	p<0.0001	p<0.0001
Δ IV–I (%)	+55%	-23%	-44%	-30%	-32%
	p<0.0001	p = 0.028	p<0.0001	p<0.000	p<0.0001

### Factors associated with late ART initiation and with advanced disease

The likelihood of late ART initiation with or without advanced HIV disease decreased over time from 2006 to 2017. In the multivariate logistic regression model, other factors associated with late ART initiation were marital status and education level. Indeed, married patients had a 1.7-fold chance of being treated late (p = 0.0007). In contrast, patients with a higher education level had a lower chance of being treated late (p = 0.0001).

There was no effect of age, gender, marital status or educational level on being initiated late with advanced disease (Table 6).

**Table 6:** Other factors associated with late initiation of ART: Multiple logistic regression models.

	Potential associated factors	Coefficient ± SE	p value	Odd ratio (95%CI)
Late ART initiation	Intercept	2.0 ± 0.46	-	-
(1 = Yes)	Age (/5 years)	0.039 ± 0.040	0.33	1.04 (0.96; 1.1)
(N = 1163)	Gender (0 = Man, 1 = Woman)	-0.062 ± 0.089	0.49	0.88 (0.62; 1.3)
	Marital status (1 = Married, 0 = No)	0.27 ± 0078	0.0007	1.7 (1.3; 2.3)
	Education level (from 0 = Illiterate to 5 = University)	-0.30 ± 0.079	0.0001	0.74 (0.64; 0.86)
ART initiated	Intercept	-0.11 ± 0.40	-	-
with advanced	Age (years)	-0.060 ± 0.034	0.075	0.94 (0.88; 1.01)
disease stage (1 = Yes)	Gender (0 = Man, 1 = Woman)	-0.099 ± 0.077	0.20	0.82 (0.61; 1.1)
(N = 1163)	Marital status (1 = Married, 0 = No)	0.11 ± 0.070	0.12	1.2 (0.94; 1.6)
	Education level (from 0 = Illiterate to 5 = University)	-0.056 ± 0.072	0.43	0.95 (0.82; 1.1)

## Discussion

To our knowledge, this study is the first to assess the impact of expanding ART eligibility criteria on the early initiation of ART in DRC since the adoption of the recommendation to treat all PLHIV regardless of the level of course of HIV infection. It retrospectively analyzed the evolutionary trends in the median CD4 count, the median time to initiation of ART, and the proportion of patients with advanced HIV disease at the initiation of ART between 2006 and 2017 and their associated factors.

This study thus covers a wide period ranging, starting when the CD4 cell count threshold for ART eligibility was 200 cells/μl to the actual strategy of treating all infected individuals. We analyzed 7,278 PLHIV medical files in 25 health centers in Kinshasa.

We observed a slow evolution toward an early initiation of ART, illustrated by a 74% increase in the median number of CD4 cells over 12 years (from 190 cells/μl in 2006 to 331/mm^3^ in 2017) as well as a significant decrease in the proportion of patients who started antiretroviral treatment late with or without advanced disease over the same period. This is due to increased access to HIV tests and earlier diagnosis and enrollment in HIV care [32]. There is also undoubtedly a great contribution of guideline implementation, which increasingly favors treating all PLHIV.

If some studies have provided encouraging results regarding early enrollment for HIV care as well as early ART initiation [33–35], other studies carried out in the context of resource-limited settings indicated that the burden of patients who initiate ART late persists [15,36–42].

The WHO estimated at the end of 2016 that approximately 30–40% of PLHIV who started ART in low- and middle-income countries had CD4 cell counts below 200 cells/mm^3^, or almost 50% in some contexts [43], with a great proportion having a CD4 cell count lower than 100 cells/mm^3^ [8,37,44]. In line with those reports, the median CD4 cell count at the initiation of ART in our study remained consistently insufficient. The median CD4 increase rate, although encouraging, remained low (11.75 cells/mm^3^/year). It would take more than 14.4 years before the median CD4 count at the start of antiretroviral therapy is > 500 cells/μl. Indeed, until 2015, we did not observe a significant increase in median CD4 levels at ART initiation. Regarding those results, our study is consistent with the data of a meta-analysis including more than 500,000 HIV-infected people in 24 countries from 2002 to 2013, which suggested that the number of CD4 cells when presenting for HIV care or at the time of initiation of antiretroviral therapy did not significantly change in sub-Saharan Africa [45].

In contrast, since 2015 and the implementation of new WHO guidelines, we observed a greater increase in CD4 cell count at ART initiation. This is in line with the results of other groups. Indeed, Nash D and Olga Tymejczyk reported, respectively, higher gains in CD4 cell count at the initiation of ART in PLHIV in 2016 and 2018 [34,35].

The trend that we observed over time thus reflects policies’ changes to faster access to treatment during the observation period of this study. The introduction of the latest "test and treat" recommendation promotes rapid initiation of antiretroviral therapy, including the offer of treatment on the day of diagnosis in the absence of clinical contraindication. It thus shortens the pretherapeutic period. Recent evidence indeed indicates that accelerated initiation procedures (the distribution of ARVs on the same day as HIV testing) can significantly increase initiation rates [46–48]. Here, we showed a significant reduction in time to ART initiation following HIV diagnosis due to the evolution of the WHO guidelines over time. However, this strategy would only partially solve the problem of late treatment initiation. Indeed, although 93% of PLHIV diagnosed in 2017 were treated within 30 days, almost half of them initiated their treatment with a CD4 cell count below 350 cells/mm^3^. The diagnosis should thus be earlier to initiate ART at an early stage of HIV infection, an issue that we described recently in another report [32]. Innovative approaches are also needed to improve the subsequent link with HIV care [49–53].

Although the 2016 WHO Consolidated ARV Guidelines recommend starting antiretroviral therapy regardless of CD4 cell count [7], clinical staging alone incurs the risk of lacking severe immune depression. In this study, 32.9% of PLHIV classified as stage 1 or 2 had a CD4 count of less than 200 cells/l (S1 Table). The CD4 cell count at the start of treatment to identify people with advanced HIV disease remains important [54–56].

In the multivariate analysis of this study, in contrast with the majority of studies of the same context, gender and age did not influence the proportion of patients treated late [27,38].

Indeed, the majority of studies conducted in resource-limited settings show that men and older subjects initiate their treatment later than women and young subjects [15,29,35,38,40,41,57–62]. This discrepancy could indicate that there are different strategies targeting these groups (men or older subjects) in place, such as community-based screening strategies, particularly among men [61].

In addition, married PLHIV are more likely to start ART late, and those with higher education are less likely to do so. Some studies show that people with a long life as a couple or a low socioeconomic level start treatment late [41,59,63,64]. A high level of education [62,65], a level of information (on HIV), and a fairly high standard of living are protective factors against the late initiation of ART [8,12,14,58–60,66].

Our results must be interpreted with the integration of certain limits.

First, several data were missing in the analyses, in particular, to some extent, the CD4 count both at diagnosis and at the initiation of treatment [67]. Missing data on several variables studied among the determinants significantly reduced the sample size in logistic regression. Second, we did not assess certain variables at the individual level that have been found to be associated with late ART initiation in other African studies, including alcohol consumption, number of sexual partners or property ownership or stigma [41,42,68–70]. Third, program and provider factors were not studied. It is likely that important and modifiable determinants other than individual factors are responsible for the results of this study.

Fourth, the sample of this study consisted of patients enrolled in urban or peri-urban HIV treatment programs. They are likely to have more opportunities or capacities than rural or semirural patients to access HIV services, including a shorter distance to the health center, a better level of HIV information, a higher level of education and a better socioeconomic level. These patients are therefore not representative of all HIV programs in the city province of Kinshasa, which also comprises rural and semirural areas.

This study also had many strengths. This study spans 12 years, covering a fairly long period during which national guidelines have been modified, in line with WHO recommendations. The data used in the analyses of this study are longitudinal data collected in routine programs and not for research purposes alone. Data were collected from a very large number of PLHIV, followed by a great number of HIV care and treatment centers in the various districts of the city province of Kinshasa.

## Conclusions

While the recommendations aim at achieving universal access to ART, it is crucial to continue to assess the evolution of ART coverage, as well as to intensify efforts to initiate rapid ART after screening for all PLHIV, whether or not they are at an advanced stage of HIV infection.

We observed an increasingly rapid initiation of ART, a slow evolution toward an early initiation of ART with a decrease in the proportion of patients treated at advanced stages of HIV disease between 2006 and 2017 in 25 establishments in the city of Kinshasa (DRC).

Despite undeniable progress, almost half of PLHIV were treated while having CD4 cell counts below 350 CD4/mm^3^ cells in 2017, although 93% started treatment within a month. Our analysis thus illustrates the persistent challenge of late ART initiation and identifies the critical need for early diagnosis. Identification of PLHIV earlier in their disease progression is imperative to ensure the benefits of ART at both the individual and population levels.

Innovative interventions or approaches, such as home and work HIV testing, HIV self-testing and screening at the point of service, are therefore needed to (i) increasingly scale up screening efforts to reach all PLHIV earlier, especially those with severe disease, (ii) promote a rapid link to HIV care and (iii) initiate ART early. Without such efforts to promote earlier initiation of treatment, the full impact of the programmatic extension of HIV control in the DRC could not be substantial in the long term.

## Supporting information

S1 Table Correlation between CD4 count and WHO stage (N = 2340 patients with CD4 count and WHO stage assessed. (DOCX)
