# Supplementary material for: Longitudinal analysis of sociodemographic, clinical and therapeutic factors of HIV-infected individuals in Kinshasa at antiretroviral therapy initiation during 2006-2017
Source: PLoS One. 2021 Nov 5;16(11):e0259073. doi: 10.1371/journal.pone.0259073 (PMC8570501; doi:10.1371/journal.pone.0259073)
Supplement: S1 Table — (DOCX) [file pone.0259073.s001.docx]

**S1 Table. Correlation between CD4 count and WHO stage (N=2340 patients with CD4 count and WHO Stage assessed**

|  | WHO stage | | | | |
| --- | --- | --- | --- | --- | --- |
|  | 1  (n=348) | 2  (n=531) | 3  (n=1328) | 4  (n=133) | Total  (n=2340) |
| CD4 (cells/mm³) |  |  |  |  |  |
| <200 | 87 (25.0) | 203 (38.2) | 729 (54.9) | 84 (63.2) | 1103 (47.1) |
| 200-350 | 99 (28.5) | 205 (38.6) | 377 (28.4) | 28 (21.0) | 709 (30.3) |
| ≥350 | 162 (46.5) | 123 (23.2) | 222 (16.7) | 21 (15.8) | 528 (22.6) |
